# Epigenome‐wide analyses identify DNA methylation signatures of dementia risk

**DOI:** 10.1002/dad2.12078

**Published:** 2020-08-10

**Authors:** Rosie M. Walker, Mairead L. Bermingham, Kadi Vaher, Stewart W. Morris, Toni‐Kim Clarke, Andrew D. Bretherick, Yanni Zeng, Carmen Amador, Konrad Rawlik, Kalyani Pandya, Caroline Hayward, Archie Campbell, David J. Porteous, Andrew M. McIntosh, Riccardo E. Marioni, Kathryn L. Evans

**Affiliations:** ^1^ Centre for Genomic and Experimental Medicine Institute of Genetics and Molecular Medicine University of Edinburgh Edinburgh UK; ^2^ MRC Centre for Reproductive Health The Queen's Medical Research Institute Edinburgh Bioquarter 47 Little France Crescent Edinburgh EH16 4TJ UK; ^3^ Division of Psychiatry University of Edinburgh Royal Edinburgh Hospital Edinburgh UK; ^4^ MRC Human Genetics Unit Institute of Genetics and Molecular Medicine University of Edinburgh Edinburgh UK; ^5^ Faculty of Forensic Medicine Zhongshan School of Medicine Sun Yat‐Sen University 74 Zhongshan 2nd Road Guangzhou 510080 China; ^6^ Division of Genetics and Genomics The Roslin Institute and Royal (Dick) School of Veterinary Studies University of Edinburgh, Easter Bush, Roslin Edinburgh UK; ^7^ Generation Scotland Centre for Genomic and Experimental Medicine Institute of Genetics and Molecular Medicine University of Edinburgh Edinburgh UK

**Keywords:** alcohol, Alzheimer's disease, dementia, DNA methylation, genetic risk score, risk factors

## Abstract

**Introduction:**

Dementia pathogenesis begins years before clinical symptom onset, necessitating the understanding of premorbid risk mechanisms. Here we investigated potential pathogenic mechanisms by assessing DNA methylation associations with dementia risk factors in Alzheimer's disease (AD)–free participants.

**Methods:**

Associations between dementia risk measures (family history, AD genetic risk score [GRS], and dementia risk scores [combining lifestyle, demographic, and genetic factors]) and whole‐blood DNA methylation were assessed in discovery and replication samples (n = ~400 to ~5000) from Generation Scotland.

**Results:**

AD genetic risk and two dementia risk scores were associated with differential methylation. The GRS associated predominantly with methylation differences in *cis* but also identified a genomic region implicated in Parkinson disease. Loci associated with dementia risk scores were enriched for those previously associated with body mass index and alcohol consumption.

**Discussion:**

Dementia risk measures show widespread association with blood‐based methylation, generating several hypotheses for assessment by future studies.

## 1 BACKGROUND

The pathophysiology of dementia begins many years, possibly decades, before the emergence of clinical symptoms.^1^ This long prodromal phase highlights the need for preventative strategies prior to the development of irreversible brain damage. As such, understanding premorbid risk mechanisms is critical. Several approaches to identify individuals at risk of developing dementia have been devised, including the summation of genetic risk, in the form of genetic risk scores (GRSs), the consideration of family history, and the calculation of risk scores, which incorporate multiple lifestyle, demographic, and genetic risk factors.^2^, ^3^, ^4^

DNA methylation is an epigenetic modification which, in some contexts, is associated with gene expression variation. Altered gene expression has been identified in the blood and post‐mortem brains of AD patients,^5^, ^6^ and post‐mortem brain‐based studies have identified associations between DNA methylation and AD and its neuropathological hallmarks.^7^, ^8^, ^9^ Blood‐based studies, while limited by small sample sizes, have also found evidence for AD‐associated methylation differences.^10^, ^11^ It is not, however, possible to determine from these studies whether methylation differences precede AD onset, making them potentially etiologically informative, or whether they reflect ongoing pathology, compensatory mechanisms and/or treatment effects. Studies that have identified associations between variation in blood‐based DNA methylation and risk factors for dementia (eg, carrying the *apolipoprotein E* (*APOE*) ε4 haplotype,^12^, ^13^ aging,^14^ and obesity^15^) suggest that the assessment of methylation in this tissue may yield insights into the pathways and processes that lead to dementia.

In this study, by assessing associations between multiple measures of dementia risk and blood‐based DNA methylation in AD‐free participants, we aim to further understand the mechanisms conferring dementia risk and characterize the role of methylation in these processes.

## 2 METHODS

### 2.1 Participants

Participants were drawn from the Generation Scotland: Scottish Family Health Study (GS:SFHS).^16^, ^17^ The cohort comprises ≈24,000 participants ≥18 years of age at recruitment. At a baseline clinical appointment, participants were phenotyped for a range of health, demographic, and lifestyle factors, and provided physical measurements and samples for DNA extraction. GS:SFHS has been granted ethical approval from the NHS Tayside Committee on Medical Research Ethics, on behalf of the National Health Service (05/S1401/89), and has Research Tissue Bank Status (15/ES/0040). GS:SFHS participants provided broad and enduring written informed consent for biomedical research.

**RESEARCH IN CONTEXT**

1. **Systematic review**: Several studies have investigated associations between DNA methylation and individual dementia risk factors (eg, aging and obesity) but none has compared multiple risk measures. We compared the methylation signatures of multifactorial dementia risk scores, an Alzheimer's disease (AD) genetic risk score (GRS), and dementia family history in the two largest single‐cohort blood‐based methylation samples.
2. **Interpretation**: In AD‐free participants, we identified methylation associations with an AD GRS and two midlife dementia risk scores, with no overlap between the GRS‐ and risk score–associated loci. The GRS analysis identified loci in *cis* to significant genome‐wide association study (GWAS) regions and a new putative AD‐risk locus, previously implicated in Parkinson disease. Loci associated with a midlife dementia risk overlapped with those associated with alcohol consumption.
3. **Future directions**: Longitudinal analyses should be performed to assess the pathogenic role of the identified loci. Analyses to assess the role of the putative novel dementia‐risk locus are warranted.

### 2.2 Calculation of dementia risk scores

**FIGURE 1:**
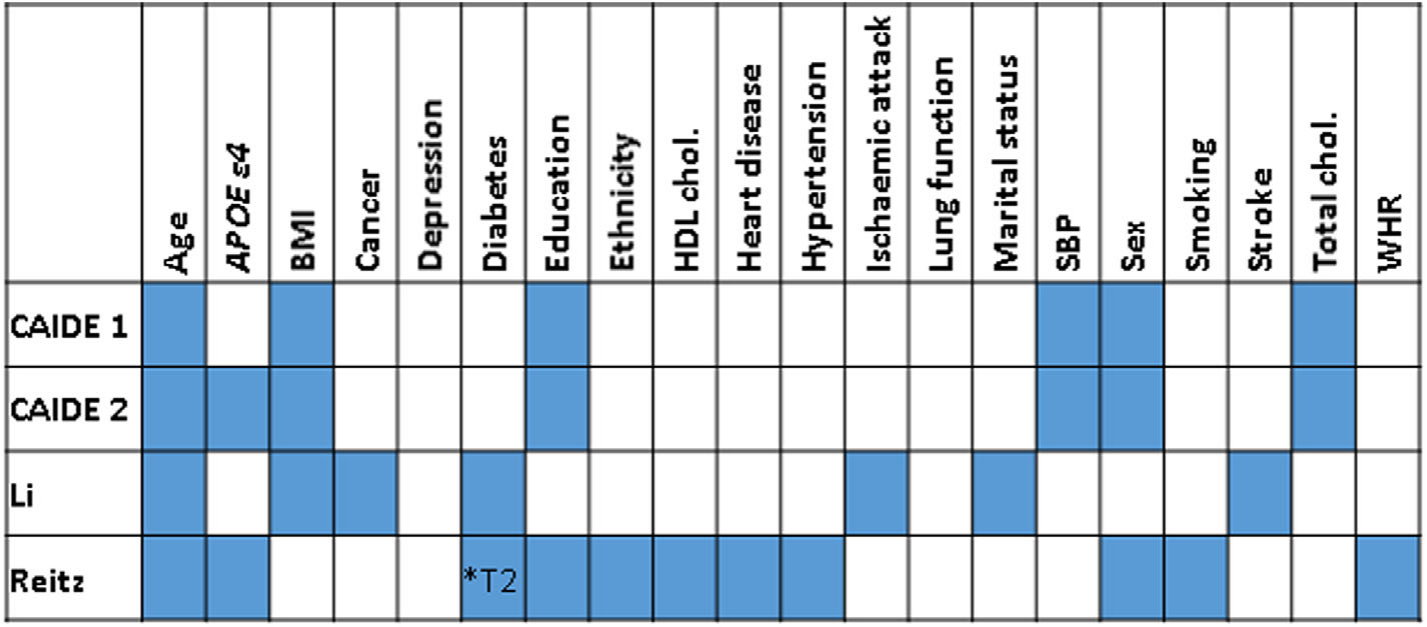
The variables contributing to each dementia risk score are indicated by filled blue boxes. Abbreviations: *APOE* ε4, apolipoprotein E ε4; BMI, body mass index; HDL, high‐density lipoprotein; chol, cholesterol; SBP, systolic blood pressure; T2, type 2; WHR, waist‐hip ratio

Four dementia risk scores, henceforth referred to as CAIDE1, CAIDE2,^2^ Li,^3^ and Reitz,^4^ were calculated using data that were collected at GS:SFHS enrollment or obtained through record linkage (see Figure 1, Supplementary Methods and Table S1 for information on the contributing variables). To generate each risk score, the contributing variables were scaled and weighted according to the original studies, and summed. The Reitz score^4^ was calculated using weightings devised when considering participants with both a “possible” and “probable” diagnosis of Alzheimer's disease (AD). Each score was calculated for participants within the appropriate age‐range (CAIDE1/2: 39‐64 years^2^; Li: ≥60 years^3^; Reitz: ≥65 years).^4^

### 2.3 Genotyping and calculation of Alzheimer's disease genetic risk score

GS:SFHS genotyping has been described previously^18^, ^19^ (Supplementary Methods). The AD GRS was calculated using the lead single‐nucleotide polymorphism (SNP) from each of the 26 genome‐wide significant loci identified through a meta‐analysis of parental AD and AD^20^ (Table S2). Each participant's score was generated by summing their dosage of each risk allele, weighted by the corresponding GWAS effect estimate.

### 2.4 DNA methylation profiling

Whole blood DNA methylation was profiled using the Infinium MethylationEPIC BeadChip (Illumina Inc.) in two sets of GS:SFHS participants at two separate times, leading to a natural discovery (n = 5190) and replication (n = 4583) design, as described previously^21^, ^22^, ^23^ (Supplementary Methods). Discovery and replication sample normalization was performed separately and the data were converted to M‐values. Participants in the replication sample were unrelated (SNP‐based relatedness <0.05) to each other and/or those in the discovery sample. A correction for relatedness was applied to the discovery sample (Supplementary Methods).

Prior to analyses, poor‐performing probes (Supplementary Methods), sex chromosome probes, participants with unreliable self‐report data, suspected XXY genotype, or self‐reported AD (n = 5) were excluded. The final discovery data set comprised 777,193 loci in 5087 participants; the replication data set comprised 773,860 loci in 4450 participants. Subsequent analyses of the methylation data were carried out using R versions 3.6.0. or 3.6.1.^24^

### 2.5 Epigenome‐wide association studies

Epigenome‐wide association studies (EWASs) were performed using linear regression modeling, implemented in limma.^25^ CpG sites (M‐values) were modeled as the dependent variable and the dementia risk measure was the predictor‐of‐interest. Additional covariates included in the standard models are detailed below:

#### 2.5.1 Discovery sample

CpG site (M‐values pre‐corrected for relatedness, estimated cell count proportions, and processing batch) ∼ dementia risk measure + age + sex + smoking status + pack years + 20 methylation principal components

#### 2.5.2 Replication sample

CpG site (M‐values) ∼ dementia risk measure+ age + sex + smoking status + pack years + estimated cell count proportions (granulocytes, natural killer cells, B lymphocytes, CD4+ T lymphocytes, and CD8+ T lymphocytes) + processing batch + 20 methylation principal components

The variables “smoking status,” “pack years,” and the methylation principal components are explained in the Supplementary Methods.

A number of sensitivity analyses for the CAIDE1 score were performed in which additional covariates were included one‐by‐one, using the same thresholds for categorizing continuous variables as implemented in the risk score. These were body mass index (BMI; ≤30 kg/m^2^ or >30 kg/m^2^); systolic blood pressure (SBP; ≤140 mm Hg or >140 mm Hg); total cholesterol (TC; ≤6.5 mmol/L or > 6.5 mmol/L); years of education (≥10, >6 and <10, or ≤6); self‐reported alcohol consumption (log_10_‐transformed (+1) units of alcohol/week), and a DNA methylation alcohol consumption score derived using the R package dnamalci.^26^, ^27^

Limma was used to calculate empirical Bayes moderated t‐statistics from which *P* values were obtained. The significance threshold in the discovery sample was *P* ≤ 3.6 × 10^−8^.^28^ Sites attaining significance in the discovery sample were assessed in the replication sample using a Bonferroni‐adjusted threshold of 0.05/no. sites assessed.

### 2.6 EWAS meta‐analysis

Inverse standard error‐weighted fixed effects meta‐analyses of the discovery and replication EWAS results were performed using METAL.^29^ Sites attaining a meta‐analysis *P* ≤ 3.6 × 10^−8^ were considered significant.

### 2.7 Identification of differentially methylated regions

Differentially methylated regions (DMRs) were identified using the dmrff.meta function in dmrff.^30^ DMRs were defined as regions containing 2 to 30 sites with consistent direction of effect and EWAS meta‐analysis *P* values ≤.05 separated by ≤500 bp. DMRs with Bonferroni‐adjusted *P* values ≤.05 were declared significant.

### 2.8 EWAS and GWAS catalog look‐ups

The GWAS Catalog v1.0.2. was downloaded from https://www.ebi.ac.uk/gwas/docs/file-downloads (December 16, 2019)^34^ and queried using gene names annotated to probes containing differentially methylated positions (DMPs) identified in the meta‐analysis (meta‐DMPs) for the phenotype‐of‐interest. GWAS traits enriched for association (*P* ≤ 1 × 10^−5^) with genes containing meta‐DMPs were identified using Fisher's exact test. Enrichment was declared significant when *P* ≤ 1.26 × 10^−5^ (0.05/3980 traits assessed).

The EWAS Catalog was downloaded from http://www.ewascatalog.org/ (03/07/19)^35^ and queried using the significant DMPs probe IDs. EWAS traits enriched for association (*P* ≤ 1 × 10^−5^) with meta‐DMPs were identified using Fisher's exact test. Enrichment was declared significant when *P* ≤ 3.31 × 10^−4^ (0.05/151 traits assessed).

### 2.9 Gene ontology/KEGG pathway analyses

Gene ontology (GO) and KEGG pathway analyses were implemented using a modified version of the missMethyl gometh function^31^ (Supplementary Methods). The target list comprised probes showing suggestive association with the phenotype‐of‐interest (*P* ≤ 1 × 10^−5^) in the EWAS or DMR analysis, and the gene universe included all probes in the analyses. Enrichment was assessed using a hypergeometric test, accounting for the bias arising from the variation in the number of probes per gene. Significance thresholds of *P* ≤ 2.20 × 10^−6^ and *P* ≤ 1.48 × 10^−4^ were applied to allow for a Bonferroni‐correction for the 22,750 GO terms and 337 KEGG pathways assessed, respectively.

### 2.10 Identification of meQTLs

Methylation quantitative trait loci (meQTLs) for the AD GRS‐associated DMPs were identified using the discovery sample. The quality control, normalization, and pre‐correction of the data prior to the meQTL analyses have been described previously^32^ (Supplementary Methods). The resulting residuals were inverse‐rank normal transformed and entered as the dependent variable in simple linear model GWASs to identify meQTLs. GWASs were implemented using REGSCAN v0.5.^33^ SNPs that were associated with a DMP with *P* ≤ 5 × 10^−8^/49 (Bonferroni correction for the 49 DMPs for which meQTL results were available), an info score ≥0.8, and had a MAF > 0.01 were declared to be meQTLs.

Where a meta‐DMP associated with AD GRS harbored a SNP at the CpG site, linkage disequilibrium between the CpG SNP and the nearest SNP contributing to the GRS was assessed using the LDpair Tool using data from the British in England and Scotland (GBR) population (https://ldlink.nci.nih.gov/?tab=ldpair; June 09, 2020).^36^

## 3 RESULTS

### 3.1 Epigenome‐wide asssociation study sample demographics

Participant numbers and sample demographic information are shown in Table S3.

### 3.2 Genetic risk for Alzheimer's disease

#### 3.2.1 Identification of differentially methylated positions

**FIGURE 2:**
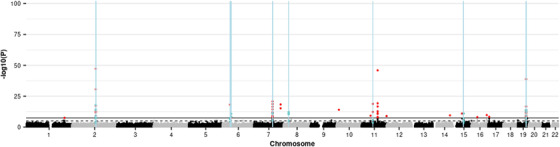
Manhattan plot showing the results of the epigenome‐wide association study (EWAS) meta‐analysis of the Alzheimer's disease (AD) genetic risk score (GRS), and the positions of differentially methylated regions (DMRs) identified in a meta‐DMR analysis. Each point represents one of the 772,453 loci included in the EWAS meta‐analysis, with the point's position being determined by genomic position (x‐axis) and significance in the EWAS meta‐analysis (–log_10_ *P* value; y‐axis). Sites attaining genome‐wide significance (*P* ≤ 3.6 × 10^−8^) are indicated in red and those that are involved in a significant DMR (Bonferroni‐correct *P* ≤ .05) are indicated in blue. The locations of DMRs are further indicated by vertical blue lines. The solid horizontal line is the threshold for genome‐wide significance (*P* ≤ 3.6 × 10^−8^) and the dashed line indicates a suggestive significance threshold (*P* ≤ 1 × 10^−5^)

An epigenome‐wide association study (EWAS) of the Alzheimer's disease (AD) genetic risk score (GRS) identified 32 differentially methylated positions (DMPs) in the discovery sample (1.06 × 10^−30^ ≤*P*≤ 2.22 × 10^−8^; Table S4). Of these, 31 showed replicated association (1.07 × 10^−30^ ≤ *P* ≤ 8.33 × 10^−4^; Table S5). Meta‐analysis of the discovery and replication samples identified 68 DMPs (6.15 × 10^−48^ ≤ *P* ≤ 3.45 × 10^−8^; Table 1; Table S6; Figure 2).

**TABLE 1:** Top 20 DMPs associated with the AD GRS in a meta‐analysis of the discovery and replication samples

ID	Chr.	BP^a^	Gene symbol	Effect	SE	*P*
cg10757760	2	127893054		0.0568	0.0039	6.15 × 10^−48^
cg04441687	11	85869322		0.0586	0.0041	1.12 × 10^−46^
cg26631131	19	45240591		0.0385	0.0029	1.56 × 10^−39^
cg02887598	2	127841945	*BIN1*	−0.0986	0.0085	2.51 × 10^−31^
cg19116668	7	99932089	*PMS2L1*	0.049	0.0051	1.54 × 10^−21^
cg18959616	11	85814918		0.0656	0.0072	5.85 × 10^−20^
cg02521229	11	60019236		0.0658	0.0073	1.61 × 10^−19^
cg03579757	7	100091793	*NYAP1*	0.0342	0.0038	2.33 × 10^−19^
cg16618979	7	143108841	*AC092214.10*	0.0974	0.0109	5.11 × 10^−19^
cg03526776	6	41159608	*TREML2*	−0.0402	0.0045	7.06 × 10^−19^
cg11461311	2	127782614	*RP11‐521O16.1;RP11‐521O16.2*	0.0321	0.0036	1.31 × 10^−18^
cg00436254	2	127862614	*BIN1*	0.0256	0.003	6.76 × 10^−18^
cg06750524	19	45409955	*APOE*	0.05	0.0059	2.18 × 10^−17^
cg23423086	11	85856245		−0.0365	0.0043	2.37 × 10^−17^
cg22906224	7	99728672	*AC073842.19*	−0.0392	0.0047	3.55 × 10^−17^
cg05908241	7	143109367	*AC092214.10*	0.0283	0.0035	6.62 × 10^−16^
cg17830204	7	99819110	*GATS;PVRIG;STAG3;AC005071.1*	0.0308	0.0039	4.78 × 10^−15^
cg19590598	2	127782813	*RP11‐521O16.1;RP11‐521O16.2*	0.0282	0.0036	5.22 × 10^−15^
cg08871934	10	11720283		−0.0343	0.0044	9.59 × 10^−15^
cg09555818	19	45449301	*APOC2;APOC4*	−0.0498	0.0065	1.24 × 10^−14^

Abbreviations: BP, base position; Chr., chromosome; SE, standard error, DMP; differentially methylated position; GRS, genetic risk score.

a Base position in genome assembly hg19/GRCh37.

Sixty‐one of the 68 meta‐DMPs were located within 18 of the 26 genome‐wide association study (GWAS) loci used to produce the GRS,^20^ with six of the remaining seven being located within 30 kb of one. Four of the associated CpGs have a single nucleotide polymorphism (SNP) at the CpG site. Density plots of the signal at these sites indicated a potential SNP effect on methylation at three of the CpGs (Figure S1). All three of these SNPs are in high linkage disequilibrium with the nearest SNP included in the GRS (D’ = 1 for cg02887598 and cg16618979, and D’ = 0.64 for cg12568536). Formal methylation quantitative trait loci (meQTL) analysis could be performed for 49/68 DMPs: of these, 48 were associated in *cis* but not *trans*, with genetic variants located in the GWAS loci.^20^ Methylation at the remaining DMP, cg14354618, which is not located within or in proximity (<30 kb) to a GWAS locus, was associated in *trans* with genetic variation on chromosome 19 (chr19: 868083‐1188756; hg19/GRCh37), which overlaps a GWAS locus.^20^

There was no overlap between the meta‐DMPs identified as being associated with the CAIDE1 score and the GRS. To explore the reason for this lack of overlap, the Pearson correlation coefficient was calculated between the two scores. This was small and non‐significant (*r* = −.018, *P* = .326).

Querying the GWAS catalog^34^ with the 34 gene names annotated to the 68 meta‐DMPs unsurprisingly identified many terms related to AD and its neuropathological hallmarks (Table S7), the most significant being “AD or family history of AD” (*P *= 1.77 × 10^−27^). No significant enrichment was identified when querying the EWAS catalog; however, this catalogue comprises results from studies using the 450K array on which only 30/68 meta‐DMPs were measured.

#### 3.2.2 Identification of differentially methylated regions

The differentially methylated region (DMR) meta‐analysis identified 18 significant DMRs comprising 41 CpGs, of which 20 were identified by the meta‐EWAS (Table S8; Figure 2). Seventeen of the DMRs overlap with loci that were in the GWAS,^20^ and the 18th is located <7 kb from the nearest GWAS locus. The longest DMR spans a 302 bp region ≈13.7 kb upstream of *BIN1*, whereas the most significant spans a 199 bp region ≈22.8 kb downstream of *BIN1*. Both show increased methylation with increased GRS.

Gene ontology (GO) analysis using the combined DMP and DMR results identified 18 terms, the most significant of which was “amyloid‐beta formation” (*P *= 3.68 × 10^−10^; Table 2). No significant KEGG pathways were identified.

**TABLE 2:** GO terms showing significant enrichment for probes where methylation is associated the AD GRS

Ontology	Term	Proportion^a^	*P*
BP	Amyloid beta formation	6/34	3.68 × 10^‐10^
BP	Negative regulation of amyloid precursor protein catabolic process	5/16	4.62 × 10^‐10^
BP	Amyloid precursor protein catabolic process	6/44	1.89 × 10^‐9^
BP	Amyloid‐beta metabolic process	6/47	2.88 × 10^‐9^
BP	Regulation of amyloid‐beta formation	5/28	1.12 × 10^‐8^
BP	Amyloid precursor protein metabolic process	6/63	1.66 × 10^‐8^
CC	Protein‐lipid complex	6/39	2.53 × 10^‐8^
BP	Regulation of amyloid precursor protein catabolic process	5/35	3.52 × 10^‐8^
BP	Negative regulation of amyloid‐beta formation	4/13	3.79 × 10^‐8^
BP	Reverse cholesterol transport	4/20	8.38 × 10^‐8^
BP	Protein‐lipid complex subunit organization	6/49	1.38 × 10^‐7^
CC	High‐density lipoprotein particle	5/26	2.42 × 10^‐7^
BP	Chylomicron remnant clearance	3/9	1.12 × 10^‐6^
BP	Triglyceride‐rich lipoprotein particle clearance	3/9	1.12 × 10^‐6^
BP	Protein‐lipid complex assembly	6/32	1.13 × 10^‐6^
CC	Plasma lipoprotein particle	6/37	1.34 × 10^‐6^
CC	Lipoprotein particle	5/37	1.34 × 10^‐6^
BP	Very‐low‐density lipoprotein particle remodeling	3/12	2.10 × 10^‐6^

Abbreviations: BP, biological process; CC, cellular component; GRS, genetic risk score; GO, gene ontology.

a Number of significant target list‐associated Entrez IDs associated with the gene ontology term/total number of Entrez IDs associated with the GO term. The target list comprised probes that met a nominal threshold for association with the AD GRS of *P* ≤ 1 × 10^−5^

### 3.3 Mid‐life dementia risk scores

The CAIDE1 and CAIDE2 risk scores assess the risk of developing dementia in 20 years' time in individuals 39 to 64 years of age.^2^ CAIDE2 takes into account the same risk factors as CAIDE1 (with different weightings) and also considers *apolipoprotein E* (*APOE)* ε4 carrier status.

#### 3.3.1 Identification of differentially methylated positions

**FIGURE 3:**
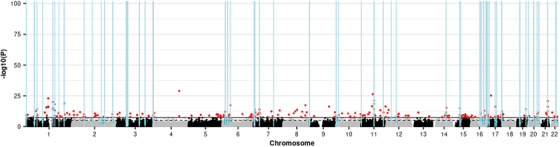
Manhattan plot showing the results of the epigenome‐wide association study (EWAS) meta‐analysis of the CAIDE1 dementia risk score and the positions of differentially methylated regions (DMRs) identified in a meta‐DMR analysis. Each point represents one of the 772,453 loci included in the EWAS meta‐analysis, with the point's position being determined by genomic position (x‐axis) and significance in the EWAS meta‐analysis (–log_10_ *P* value; y‐axis). Sites attaining genome‐wide significance (*P* ≤ 3.6 × 10^−8^) are indicated in red and those that are involved in a significant DMR (Bonferroni‐correct *P* ≤ .05) are indicated in blue. The locations of DMRs are further indicated by vertical blue lines. The solid horizontal line is the threshold for genome‐wide significance (*P* ≤ 3.6 × 10^−8^) and the dashed line indicates a suggestive significance threshold (*P* ≤ 1 × 10^−5^)

An EWAS of the CAIDE1 score in the discovery sample identified 76 DMPs (3.29 × 10^−20^ ≤*P*≤ 3.49 × 10^−8^; Table S9), of which 65 replicated (7.76 × 10^−18^ ≤ *P* ≤ 6.48 × 10^−4^; Table S10). Meta‐analysis of the discovery and replication samples identified 227 DMPs (1.20 × 10^−29^ ≤ *P* ≤ 3.58 × 10^−8^; Figure 3; Table 3; Table S11).

**TABLE 3:** Top 20 DMPs associated with the CAIDE1 risk score in a meta‐analysis of the discovery and replication samples

ID	Chr.	BP	Gene symbol	Direction	effect	SE	*P*
cg06690548	4	139162808	*SLC7A11*	–	−0.0766	0.0068	1.20 × 10^‐29^
cg19758958	11	62319222	*AHNAK*	–	−0.0256	0.0024	4.22 × 10^‐27^
cg11024682	17	17730094	*SREBF1*	++	0.026	0.0025	5.31 × 10^‐26^
cg14476101	1	120255992	*PHGDH*	–	−0.0391	0.0039	1.14 × 10^‐23^
cg00574958	11	68607622	*CPT1A*	–	−0.0413	0.0043	8.30 × 10^‐22^
cg06500161	21	43656587	*ABCG1*	++	0.0225	0.0024	2.10 × 10^‐21^
cg19693031	1	145441552	*TXNIP*	–	−0.0369	0.004	8.74 × 10^‐21^
cg22699725	1	207242586	*PFKFB2*	++	0.0275	0.003	6.89 × 10^‐20^
cg00683922	1	207242569	*PFKFB2*	++	0.0272	0.003	2.70 × 10^‐19^
cg05325763	11	68607719	*CPT1A*	–	−0.0394	0.0044	2.90 × 10^‐19^
cg22976567	1	156074182	*LMNA*	–	−0.026	0.0029	7.06 × 10^‐19^
cg02715788	8	119974400		–	−0.0225	0.0026	4.44 × 10^‐18^
cg18120259	6	43894639	*LOC100132354*	–	−0.0209	0.0024	4.49 × 10^‐18^
cg11376147	11	57261198	*SLC43A1*	–	−0.0236	0.0028	2.98 × 10^‐17^
cg00163198	11	130767760	*SNX19*	++	0.0237	0.0028	4.89 × 10^‐17^
cg16246545	1	120255941	*PHGDH*	–	−0.0255	0.003	5.16 × 10^‐17^
cg01270753	9	101944336	*RP11‐96L7.2*	–	−0.034	0.0041	5.27 × 10^‐17^
cg08857797	17	40927699	*VPS25*	++	0.0236	0.0028	5.86 × 10^‐17^
cg16740586	21	43655919	*ABCG1*	++	0.0243	0.0029	7.52 × 10^‐17^
cg26457483	1	120256112	*PHGDH*	–	−0.0308	0.0037	1.76 × 10^‐16^

Abbreviations: BP, base position; Chr., chromosome; SE, standard error; DMP, differentially methylated position.

^a^Base position in genome assembly hg19/GRCh37.

An EWAS of the CAIDE2 score in the discovery sample identified 18 DMPs (1.96 × 10^−17^ ≤*P*≤ 3.24 × 10^−8^; Table S12), of which 17 replicated (4.91 × 10^−12^ ≤ *P* ≤ 2.01 × 10^−3^; Table S13). Meta‐analysis of the discovery and replication samples identified 59 DMPs (3.56 × 10^−22^ ≤ *P* ≤ 3.21 × 10^−8^; Table S14). Fifty‐four of the CAIDE2 meta‐DMPs were also identified by the CAIDE1 meta‐analysis; given this overlap, subsequent analyses focus on CAIDE1.

The CAIDE1‐associated meta‐DMPs were explored using the EWAS and GWAS catalogs.^34^ Significant enrichment was identified for 16 EWAS traits/conditions, with “Body mass index [BMI]” being the most significantly enriched (*P *= 2.96 × 10^−118^; Table S15). Two alcohol‐related traits: “alcohol consumption per day” (*P *= 9.70 × 10^−29^) and “gamma‐glutamyl transferase” (*P *= 8.55 × 10^−25^) also showed enrichment. GWAS catalog enrichment analysis identified only one significant term, “Eosinophil counts” (*P *= 4.97 × 10^−8^).

##### Sensitivity analyses

The extent to which the BMI component of the CAIDE1 score drives the observed CAIDE1 associations was assessed by performing an EWAS meta‐analysis in which BMI was included as an additional covariate. Co‐varying for BMI resulted in only 11 of the original 227 meta‐DMPs remaining significant (Table S11), with the correlation between the effect estimates for all sites between the two analyses being *r *= 0.745 (95% confidence interval (CI) = 0.744 to 0.746). Compared with BMI, co‐varying for other components of the CAIDE1 score resulted in a larger numbers of CAIDE1 meta‐DMPs remaining significant (SBP: 94/227; TC: 190/227; education: 221/227; Table S11); correlations with the effect estimates of the original analysis were higher (SBP: *r *= 0.815, 95% CI = 0.815 to 0.816; TC: *r *= 0.938, 95% CI = 0.937 to 0.938); education: *r *= 0.990, 95% CI = 0.990 to 0.990).

##### Assessment of the involvement of alcohol consumption in the CAIDE1 EWAS results

Twenty‐four of the 88 meta‐DMPs that are represented on the 450K array, including the most significant DMP, cg06690548, have previously been associated with alcohol consumption (*P* ≤ 1 × 10^−5^).^26^ Similarly, an EWAS meta‐analysis of self‐reported alcohol consumption in our methylation sample identified 5599 DMPs at a suggestive threshold of *P* ≤ 1 × 10^−5^ (unpublished data, Clarke et al.); of these, 49 show a significant association with CAIDE1 with a consistent direction of effect. This overlap is highly significant (*P *< 2 × 10^−16^).

Because alcohol consumption showed a small but significant correlation with CAIDE1 score (*r *= 0.091; 95% CI = 0.065 to 0.118; *P *= 2.60 × 10^−11^), the potential for alcohol consumption to drive the observed associations between CAIDE1 and DNA methylation was assessed by including alcohol consumption measured by (1) self‐report or (2) a polyepigenetic risk score^26^, ^27^ as an additional covariate in the CAIDE1 EWAS. Neither measure of alcohol consumption resulted in a substantial change in effect estimates (self‐reported: *r *= 0.948, 95% CI = 0.948 to 0.948; DNA methylation score: *r* = 0.993, 95% CI = 0.993 to 0.993), with 166 and 191 of the 227 CAIDE1 meta‐DMPs remaining significant after the inclusion of the self‐reported and DNA methylation score coefficients, respectively (Table S11).

#### 3.3.2 Identification of differentially methylated regions

The DMR meta‐analysis of the discovery and replication samples identified 57 CAIDE1‐associated DMRs (all Bonferroni‐adjusted *P *< 0.044; Table S16), each comprising two to seven CpGs. In total, the 57 DMRs involve 179 sites, of which 35 were significant in the EWAS meta‐analysis. The most significant DMR (Bonferroni‐adjusted *P *= 1.67 × 10^−20^) comprises four hypomethylated CpGs spanning a 115 bp intronic region of *CPT1A* (chr11: 68607622‐68607737; hg19/GRCh37). The longest DMR spans a 1.1 kb intronic region of *JARID2* (chr6: 15504844‐15505949; hg19/GRCh37).

GO and KEGG pathway analyses found no enrichment for biological processes or pathways among the CAIDE1 DMP or DMR CpG sites (min. *P*_GO _= 8.46 × 10^−4^; minutes. P_KEGG _= 3.51 × 10^−3^).

### 3.4 Other measures of dementia risk

The other measures of dementia risk assessed were (1) dementia family history (FH) and (2) two late‐life dementia risk scores that predict the risk of developing dementia in those older than 60 or 65 years of age.^3^, ^4^ EWASs of the discovery sample (minimum (min). *P*_FH _= 8.47 × 10^−7^; min. *P*_Li _= 3.91 × 10^−8^; min *P*_Reitz _= 1.58 × 10^−6^), meta‐EWASs of the discovery and replication samples (min. *P*_FH _= 1.15 × 10^−6^; min. *P*_Li _= 1.80 × 10^−6^; min. *P*_Reitz _= 8.99 × 10^−7^), and DMR analyses (min. Bonferroni‐adjusted *P*_FH _= 0.439; min. Bonferroni‐adjusted *P*_Li _= 0.208; min. Bonferroni‐adjusted *P*_Reitz _= 1) failed to identify any significant associations.

## 4 DISCUSSION

We have assessed DNA methylation associations with a range of dementia risk measures in large discovery and replication samples comprising Alzheimer's disease (AD)‐free participants, and we report multiple loci as being associated with AD genetic risk and two multifactorial mid‐life risk scores for dementia.

All but one of the loci associated with the AD genetic risk score (GRS) were located within 30 kb of the genome‐wide association study (GWAS) loci used to derive the GRS,^20^ with methylation quantitative trait loci (meQTL) analysis supporting involvement of *cis* meQTLs. Only one differentially methylated position (DMP), cg14354618 on chromosome 11, was an exception to this pattern, being associated with *trans* meQTLs in a GWAS risk locus on chromosome 19. cg14354618 is located in a CpG island in *AP001979.1*. Genetic variation annotated to *AP001979*.1 has been associated with Parkinson's disease,^37^, ^38^ body fat percentage,^39^ and sugar consumption^40^ but has not been associated with AD in large‐scale GWASs.^41^, ^42^ There is a degree of overlap between the clinical features and pathologies associated with AD and Parkinson's disease, with certain genetic variants being associated with both.^43^, ^44^ Moreover, obesity and hyperglycemia have been implicated as dementia risk factors.^45^ Taken together, these findings suggest this locus to be a plausible AD‐risk locus, which warrants further investigation.

Considering both the meta‐DMP and differentially methylated region (DMR) results, two regions harbor a large number of AD GRS‐associated sites. These regions contain (1) *BIN1* and (2) *PVRL2*, *APOE*, *APOC4*, and *APOC2* (henceforth referred to as the *APOE* locus). The *APOE* locus has not been identified previously by brain‐based epigenome‐wide association studies (EWASs) of AD neuropathological hallmarks^7^, ^46^ or a blood‐based AD case‐control EWAS^44^; larger samples might be required to detect association between methylation at this locus and AD.

In contrast, several studies have identified altered methylation of *BIN1* in AD patients or in association with AD neuropathological hallmarks.^7^, ^47^ ^,^ ^48^ These findings are of particular interest, as altered *BIN1* brain expression has been reported in AD^49^, ^50^, ^51^ and DNA methylation has been suggested to regulate *BIN1*’s expression.^52^ We identified a mixture of hyper‐ and hypomethylation in the upstream region and gene body and hypermethylation in the downstream region. Although none of the identified sites directly replicated those identified by previous studies, it is noteworthy that one of our hypermethylated meta‐DMPs (cg18813565) is located only 31 bp from a site (that failed our quality control) at which increased methylation in the dorsolateral prefrontal cortex has been associated with neuritic plaque burden and AD diagnosis.^47^ Moreover, this site contributes to a hypermethylated DMR that spans a 199 bp region located ≈23 kb downstream of *BIN1*. This region overlaps with non‐coding RNAs, RP11‐521O16.1, and RP11‐521O16.2, suggesting the possibility that altered methylation of these non‐coding RNAs might alter *BIN1* expression. This hypothesis should be assessed by future studies.

The CAIDE1 risk score is a composite score formed by the weighted summation of age, sex, body mass index (BMI), years in education, systolic blood pressure, and total cholesterol.^2^ It is designed for the prediction of dementia in 20 years' time in individuals 39 to 64 years of age, which it does with an area under the curve of 0.77 (95% confidernce interval 0.71 to 0.83). Because age and sex were covariates in our analytical models, the differential methylation observed in this study reflects the modifiable “lifestyle” components of the score. Our analyses revealed BMI to be the primary driver of the CAIDE1‐associated methylation differences. We identified significant overlap between the sites associated with CAIDE1 and those that have been associated previously with alcohol consumption. Strikingly, the most significant CpG in our analysis of CAIDE1 was also the most significant CpG in a recent Generation Scotland: Scottish Family Health Study (GS:SFHS) alcohol consumption EWAS (unpublished data, Clarke et al.). Although alcohol consumption was significantly correlated with CAIDE1 score, it could not account for the CAIDE1‐associated differences in methylation. This finding is of interest in light of the observed associations between excessive alcohol consumption and dementia risk.^53^ Our findings suggest that the risk factors contributing to the CAIDE1 score and alcohol consumption might confer risk for dementia via independent effects on common pathways.

The loci implicated by our analyses of the AD GRS and the CAIDE1 score did not overlap, and they did not implicate common genes. In keeping with this, the correlation between the scores was small and non‐significant. This lack of concordance might be attributable to differences in the methodology used to create the scores: although the CAIDE1 score was trained using a sample comprising mixed dementia cases (of which ≈75% were diagnosed with AD),^2^ the AD GRS was devised using a sample comprising AD and proxy AD cases.^20^ Moreover, the CAIDE 1 score predominantly comprises cardiovascular risk factors for dementia, meaning that it is likely to identify a sub‐population of those at risk for dementia.

We did not observe any DNA methylation associations with AD family history (FH) or two late‐life dementia risk scores. The lack of associations with AD FH is somewhat surprising, as this has been shown previously to be a good AD proxy‐phenotype.^20^ Our failure to observe significant associations for these traits may reflect a lack of statistical power, particularly as the samples available for the late‐life dementia risk scores were relatively small.

It is important to note some additional strengths and limitations to our study. Although it would clearly be desirable to study DNA methylation in brain tissue, growing evidence highlights the contribution of systemic factors to dementia pathogenesis.^54^ Thus methylation studies in the blood are necessary to provide a holistic characterization of the processes that contribute to dementia development. Moreover, profiling blood methylation permits both longitudinal analyses to characterize the dynamic processes underlying dementia pathogenesis and biomarker identification.

An important limitation of our study is that the use of a cross‐sectional design means that causal inferences cannot be drawn. A corollary of this is that it is difficult to determine whether the methylation differences assessed play a causal role in the development of dementia. In some cases, causal inference analyses to assess relationships with important intermediary variables such as cognitive ability and cognitive decline together with Mendelian randomization may help delineate likely causality; future studies should assess this possibility.

Ultimately, the longitudinal assessment of cognitive decline and the development of dementia will also be necessary to address questions about causation. Moreover, the availability of longitudinal data would also permit the development of an epigenetic (and potentially a multifactorial genetic, epigenetic, and lifestyle factors) predictor of dementia. An important conceptual issue that must be considered when attempting to determine causality from longitudinal data is that that the pathogenesis of dementia is itself a gradual process involving quantitative changes in multiple biological systems, which eventually result in the binary diagnosis of dementia. As such, it might not be possible to strictly delineate the temporal relationship between risk factors, their biological correlates and the onset of dementia. Instead, the identification of co‐occurring processes might yield experimentally testable hypotheses. An additional factor to consider is that the non‐genetic risk factors that contribute to the scores assessed are themselves only associated with the development of dementia and do not necessarily play a causal role. Future studies that aim to delineate the causal contribution of these factors to dementia will facilitate the development of risk scores whose primary purpose is for use in the investigation of pathogenic mechanisms.

Other limitations concern the quality of the variables used in the dementia risk scores: self‐reporting may have resulted in errors, and the blood samples used for cholesterol measurements were not taken at a consistent time of day or after a consistent fasting length. Furthermore, we considered only a sub‐set of putative dementia risk factors. The demographic and lifestyle risk factors considered in this study were selected due to their involvement in validated composite risk scores;^2^, ^3^, ^4^ however, it would be of interest to examine DNA methylation associations of other well‐supported dementia risk factors in future studies.

Here we present the first comprehensive characterization of associations between blood DNA methylation and dementia risk, performed in the largest single‐cohort methylation samples collected to date. We identify several CpGs where methylation is associated with dementia risk measures and identify a putative novel AD risk locus. Our findings suggest a number of hypotheses for assessment by future studies, which should include longitudinal assessments of the causal nature of methylation in dementia pathogenesis.

## CONFLICTS OF INTEREST

Andrew M. McIntosh has received grant support from Pfizer, Eli Lilly, Janssen, and The Sackler Trust. These sources are not connected to the current investigation. Andrew M. McIntosh has also received speaker's fees from Janssen and Illumina. The remaining authors report no conflicts of interest.

## Supporting information

Supplementary Information

Supplementary Information

Supplementary Information

Supplementary Information

Supplementary Information

Supplementary Information

Supplementary Information

Supplementary Information

Supplementary Information

Supplementary Information

Supplementary Information

Supplementary Information

Supplementary Information

Supplementary Information

Supplementary Information

Supplementary Information

Supplementary Information

Supplementary Information
