# Supplementary material for: Epigenome‐wide analyses identify DNA methylation signatures of dementia risk
Source: Alzheimers Dement (Amst). 2020 Aug 10;12(1):e12078. doi: 10.1002/dad2.12078 (PMC7416667; doi:10.1002/dad2.12078)
Supplement: Supplementary file 1 — Supplementary Information [file DAD2-12-e12078-s001.docx]

| **Variable** | **Details of measurement** |
| --- | --- |
| Age | Calculated from self-reported date of birth and the date of the GS:SFHS clinical appointment. |
| *APOE* ε4 | Inferred from genotyping of rs429358 and rs7412 using TaqMan probes. |
| BMI | Calculated from height (cm) and weight (kg) measured during the clinical appointment. |
| Bowel cancer | Self-report of ever being affected |
| Breast cancer | Self-report of ever being affected |
| Depression | Participants who answered “yes” to either of two screening questions (“Have you ever seen anybody for emotional or psychiatric problems?” and “Was there ever a time when you, or someone else, thought you should see someone because of the way you were feeling or acting?”) were assessed for MDD using the SCID. Participants were deemed not to have depression if they answered the two screening questions negatively. |
| Diabetes | Defined using Scottish Care Information Diabetes Collaboration (SCI-DC) records (<http://www.sci-diabetes.scot.nhs.uk/>). Participants were considered to have diabetes if they were recorded as having Type 1 or Type 2 diabetes (Li) or just Type 2 diabetes (Reitz) at the time of their clinical appointment. Participants were set as missing if they were recorded as having another type of diabetes. |
| Diastolic blood pressure | Measured during clinical appointment using an Omron BP Monitor |
| Education | Self-reported answer to “How many years altogether did you attend school or study full-time?”. Two versions of the GS:SFHS questionnaire were used. The first version required an answer in years, whilst the second version permitted the following responses: 0, 1-4, 5-9, 10-11, 12-13, 14-15, 16-17, 18-19, 20-21, 22-23, 24+. To combine the data from the two questionnaires, the midpoint of the categories was used for the second version of the questionnaire. Another question asked participants about the highest educational qualification they had achieved. Participants for whom the answer to the two education questions were incompatible were set as missing. |
| Ethnicity | Self-report |
| HDL cholesterol | Measured in serum obtained during clinical appointment |
| Heart disease | Self-report of ever being affected |
| Ischaemic attack | Self-report of ever having a stroke |
| Lung cancer | Self-report of ever being affected |
| Lung function | Forced expiratory volume measured during clinical appointment |
| Marital status | Self-report of living “as a couple” |
| Prostate cancer | Self-report of ever being affected |
| Systolic blood pressure | Measured during clinical appointment using an Omron BP Monitor |
| Sex | Self-report |
| Smoking | Self-reported answer to “Have you ever smoked tobacco?”. Possible answers were “Yes, currently smoke”, “Yes, but stopped within the past 12 months”, “Yes, but stopped more than 12 months ago”, “No, never smoked” |
| Stroke | Self-report of ever being affected |
| Total cholesterol | Measured in serum obtained during clinical appointment |
