# Supplementary figures and images for: Epigenome‐wide analyses identify DNA methylation signatures of dementia risk
Source: Alzheimers Dement (Amst). 2020 Aug 10;12(1):e12078. doi: 10.1002/dad2.12078 (PMC7416667; doi:10.1002/dad2.12078)

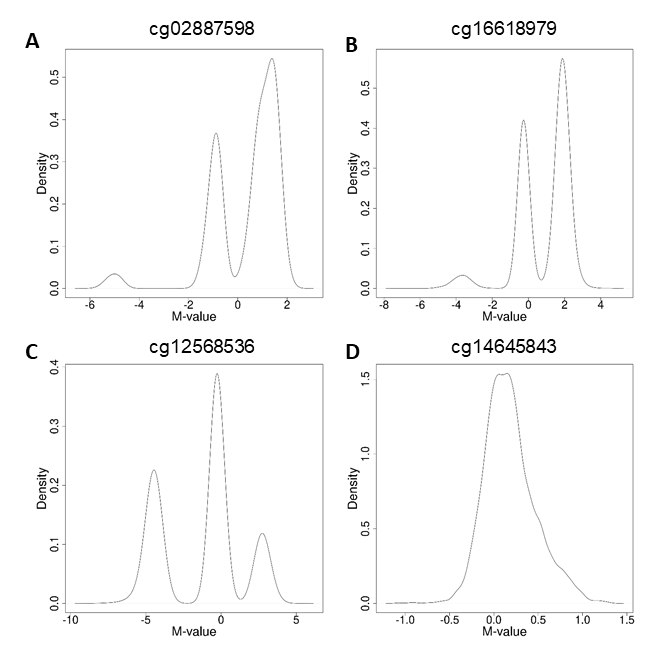

Supplement: Supplementary file 17 — Supplementary Information [file DAD2-12-e12078-s012.tif]
