# Supplementary material for: Epigenome‐wide analyses identify DNA methylation signatures of dementia risk
Source: Alzheimers Dement (Amst). 2020 Aug 10;12(1):e12078. doi: 10.1002/dad2.12078 (PMC7416667; doi:10.1002/dad2.12078)
Supplement: Supplementary file 18 — Supplementary Information [file DAD2-12-e12078-s018.docx]

**Supplementary methods**

**Epigenome-wide analyses identify DNA methylation signatures of dementia risk**

Rosie M. Walker, Mairead L. Bermingham, Kadi Vaher, Stewart W. Morris, Toni-Kim Clarke, Andrew D. Bretherick, Yanni Zeng, Carmen Amador, Konrad Rawlik, Kalyani Pandya, Caroline Hayward, Archie Campbell, David J. Porteous, Andrew M. McIntosh, Riccardo E. Marioni, Kathryn L. Evans

1. **Calculation of dementia risk scores**
   1. **CAIDE scores**

The CAIDE 1 and 2 scores, devised by Kivipelto et al. 2006 [1] are designed for use between 39 and 64 years of age. The contributing variables and their weights (in parentheses) are: age in years (≤47 (0), 47-53 (1.084), or >53 (1.762)); education in years (≥10 (0), 7-9 (0.910), 0-6 (1.281)); sex (female (0), male (0.470); systolic blood pressure (≤140 mm Hg (0), >140 mm Hg (0.791)); body mass index (≤30 kg/m2 (0), >30 kg/m2 (0.631)); total cholesterol (≤6.5 mmol/L (0), >6.5 mmol/L (0.631)); physical activity (active (0), inactive (0.527)); and, for CAIDE 2 only, *APOE* ε4 status (non-ε4 (0), ε4 (0.890)). As Kivipelto et al. did not find physical activity to be a significant predictor of dementia, it was not included when calculating these scores for GS:SFHS participants.

- 1. **Li Score**

Li et al. (2018) [2] devised a dementia risk score for use in those aged 60 years or over. The contributing variables and their weights (in parentheses) are: age in years (60-69 (0), 70-79 (1.1091), ≥80 (2.0881)); marital status (single (0.2721), married (0), formerly married (0.1713)); BMI (<18.5 kg/m^2^ (0.6659), 18.5-25 kg/m^2^ (0), >25 (0.1484)); stroke (no (0), yes (0.8204)); diabetes (no (0), yes (0.3484)); ischaemic attack (no (0), yes (0.6961)); cancer (no (0), yes (0.2872)).

- 1. **Reitz score**

Reitz et al. (2010) [3] developed a dementia risk score for use in those aged 65 years or over. The contributing variables and their weights (in parentheses) are: sex (male (0), female (0.14)); age in years (65-70 (0), >70-75 (0.809), >75-80 (1.12), >80-85 (1.862), >85 (2.892)); diabetes (no (0), yes (0.461)); hypertension (no (0), yes (0.147)); current smoking (no (0), yes (0.684)); low HDL cholesterol (no (0), yes (0.47)); high waist-to-hip ratio (no (0), yes (0.967)); education in years (>9 (0), 7-9 (1.103), 0-6 (1.506)); ethnicity (white (0), black (0.664), Hispanic (0.522)), *APOE* ε4 allele count (none (0), ≥1 (0.604)). Hypertension was defined as values above 140 mm Hg (systolic) and 90 mm Hg (diastolic); participants were deemed not to have hypertension if they did not meet these criteria. A high waist-to-hip ratio was defined as > 0.9 in males and > 0.85 in females. As Reitz et al. did not define a threshold for low HDL cholesterol, it was defined using the following standard thresholds: HDL-C<1 mmol/L (females) or <0.9 mmol/L (males).

1. **Genome-wide DNA methylation profiling**

Whole blood genomic DNA (500ng) from 9,778 participants was treated with sodium bisulphite using the EZ-96 DNA Methylation Kit (Zymo Research, Irvine, California), following the manufacturer’s instructions. DNA methylation was profiled using the Infinium MethylationEPIC BeadChip (Illumina Inc.), according to the manufacturer’s protocol. DNA methylation was profiled in the discovery (n = 5,190) and replication (n = 4,588) samples at separate time points and quality control and normalisation of the samples was carried out separately. R version 3.3.2 [4] was used for the discovery sample and 3.4.3 [5] for the replication sample. These steps have been described in detail previously [6-8]; however, briefly, outlier sites and participants, together with participants for whom there was a mismatch between their predicted sex (based on DNA methylation data) and their recorded sex, were excluded from both samples.

1. **Pre-processing of the methylation data prior to EWAS and DMR analyses**

The discovery and replication samples were then normalised (separately) using the dasen method from the wateRmelon R package [9] and converted to M-values using the beta2m function in lumi [10].

As the discovery sample included related participants, the M-values for CpGs on autosomal chromosomes in this sample were pre-corrected for relatedness, estimated white blood cell proportions and processing batch using DISSECT [11]. This was achieved by saving the residuals from a mixed linear model that included methylation as the dependent variable and the following predictor variables: a genetic relatedness matrix fitted in a leave-one-chromosome-out fashion (i.e. SNPs on the same chromosome as the CpG were excluded); proportions of granulocytes, natural killer cells, B-lymphocytes, CD4+ T-lymphocytes and CD8+ T-lymphocytes estimated using an implementation of Houseman et al.’s [12] cell type prediction algorithm in minfi [13]; and a variable that indicated the batch in which array hybridisation, staining and scanning took place. Participants in the replication sample were selected to be unrelated (SNP-based genetic relatedness < 0.05) to each other and/or participants in the discovery sample.

Prior to use in analyses, probes that had been predicted to cross-hybridise or bind sub-optimally by McCartney et al. [14] or Zhou et al. [15] were excluded. Probes on the X or Y chromosomes were also excluded. An additional four participants were excluded from the discovery sample: three participants who had answered “yes” for all self-reported conditions and one whose methylation data indicated likely XXY genotype. The final discovery sample dataset comprised corrected M-values at 777,193 loci measured in 5,087 participants, while the replication sample dataset comprised M-values at 773,860 loci measured in 4,450 participants. Subsets of these samples were analysed in each epigenome-wide association study (EWAS). All subsequent analyses of the DNA methylation data were carried out using R versions 3.6.0. or 3.6.1. [16].

1. **Smoking-related variables**

“Smoking status” is a categorical variable with five levels (current smoker, former smoker who gave up less than 12 months ago, former smoker who gave up 12 months or more ago, never smoked, undeclared) and “pack years” is a measure that indicates an individual’s lifetime exposure to tobacco. Pack years were calculated by multiplying the years an individual had smoked for by the maximum number of packs of cigarettes they ever smoked per day (a pack = 20 cigarettes). A conversion was used for cigars (a cigar = four cigarettes) and rolling tobacco (a 25g pack = 50 cigarettes).

1. **Methylation principal components**

Methylation principal components were calculated from M-values that had been pre-corrected for age, sex, estimated cell proportions, processing batch and relatedness (discovery sample only) using the R package FactoMineR [17].

1. **Gene ontology/KEGG pathway analyses**

Gene ontology (GO) and KEGG pathway analyses were carried out using a modified version of the gometh function from the missMethyl R package [18]. The function was altered with the aim of increasing the number of EntrezIDs that probes are annotated with. The first change to the function involves annotating probes with gene names from both the UCSC and GenCode databases (“UCSC_RefGene_Name” and “GencodeCompV12_NAME” columns from the EPIC array annotation object available in the IlluminaHumanMethylationEPICanno.ilm10b4.hg19 R package [19]), rather than just the UCSC database. The alias finding step of the function was updated to search in both directions (i.e. gene symbol annotations were added to a probe by searching both the “canonical” symbols to identify alias symbols and the alias symbols to identify missing “canonical” symbols).

1. **Genotyping and imputation**

The genotyping of GS:SFHS has been described in detail previously [20, 21]. Briefly, genome-wide genotype data was generated using either the Illumina Human OmniExpressExome-8-v1.0 BeadChip or the Illumina HumanOmniExpressExome-8 v1.2 BeadChip by the Genetics Core Laboratory at the Clinical Research Facility, Edinburgh, Scotland (www.wtcrf.ed.ac.uk). Genotype data was processed using the IlluminaGenomeStudio Analysis software v.2011.1 (Illumina, San Diego, CA). Quality control was carried out to remove SNPs with: <98% call rate or a Hardy-Weinberg equilibrium *p* ≤ 1 x 10^-6^.

For the meQTL analysis, imputation was performed on 602,450 autosomal SNPs following the Sanger Imputation Service pipeline (https://imputation.sanger.ac.uk/), which uses the Haplotype Reference Consortium reference panel release 1.1 [22]. Imputed SNPs with an info score ≥ 0.8 and MAF ≥ 0.01 were used in the analyses.

1. **Identification of methylation quantitative trait loci**

MeQTLs were identified using the entire discovery sample. Following quality control, the data was normalised and corrected as described previously [23]. Briefly, normalisation was carried out using preprocessNoob in the R package minfi [13] and linear mixed modelling was used in two stages to remove effects attributable to technical factors, a genomic relationship matrix, a kinship relationship matrix, three environmental relationship matrices (full sibling relationships, couple relationships and nuclear family relationships), and age, age^2^, gender, estimated cell counts (granulocytes, B-lymphocytes, natural killer cells, CD4+ T-lymphocytes, and CD8+ T-lymphocytes), season of the visit, appointment time of the day, and appointment day of the week. Following the removal of probes that have been predicted to cross-hybridise or bind sub-optimally [14, 15], corrected data was available for 49 of the 68 Alzheimer’s disease genetic risk score-associated DMPs. The resulting residuals were inverse rank normal transformed before being entered as the dependent variable in simple linear model GWASs (implemented using REGSCAN v0.5 [24]) to identify meQTLs. SNPs that were associated with a DMP with *P*≤5 x 10-8/49 (Bonferroni correction for the 49 DMPs for which meQTL results were available) were declared to be meQTLs.

**References**

[1] Kivipelto M, Ngandu T, Laatikainen T, Winblad B, Soininen H, Tuomilehto J. Risk score for the prediction of dementia risk in 20 years among middle aged people: a longitudinal, population-based study. Lancet Neurol. 2006;5:735-41.

[2] Li J, Ogrodnik M, Devine S, Auerbach S, Wolf PA, Au R. Practical risk score for 5-, 10-, and 20-year prediction of dementia in elderly persons: Framingham Heart Study. Alzheimer's & dementia : the journal of the Alzheimer's Association. 2018;14:35-42.

[3] Reitz C, Tang MX, Schupf N, Manly JJ, Mayeux R, Luchsinger JA. A summary risk score for the prediction of Alzheimer disease in elderly persons. Arch Neurol. 2010;67:835-41.

[4] Team RC. R: A language and environment for statistical computing. Vienna, Austria: R Foundation for Statistical Computing; 2016.

[5] Team RC. R: A language and environment for statistical computing. Vienna, Austria: R Foundation for Statistical Computing; 2017.

[6] Barbu MC, Walker RM, Howard DM, Evans KL, Whalley HC, Porteous DJ, et al. Epigenetic prediction of major depressive disorder. medRxiv. 2019:19001123.

[7] Bermingham ML, Walker RM, Marioni RE, Morris SW, Rawlik K, Zeng Y, et al. Identification of novel differentially methylated sites with potential as clinical predictors of impaired respiratory function and COPD. EBioMedicine. 2019;43:576-86.

[8] Madden RA, McCartney DL, Walker RM, Hillary RF, Bermingham ML, Rawlik K, et al. Birth weight predicts psychiatric and physical health, cognitive function, and DNA methylation differences in an adult population. bioRxiv. 2019:664045.

[9] Pidsley R, CC YW, Volta M, Lunnon K, Mill J, Schalkwyk LC. A data-driven approach to preprocessing Illumina 450K methylation array data. BMC Genomics. 2013;14:293.

[10] Du P, Kibbe WA, Lin SM. lumi: a pipeline for processing Illumina microarray. Bioinformatics. 2008;24:1547-8.

[11] Canela-Xandri O, Law A, Gray A, Woolliams JA, Tenesa A. A new tool called DISSECT for analysing large genomic data sets using a Big Data approach. Nature communications. 2015;6:10162.

[12] Houseman EA, Accomando WP, Koestler DC, Christensen BC, Marsit CJ, Nelson HH, et al. DNA methylation arrays as surrogate measures of cell mixture distribution. BMC Bioinformatics. 2012;13:86.

[13] Aryee MJ, Jaffe AE, Corrada-Bravo H, Ladd-Acosta C, Feinberg AP, Hansen KD, et al. Minfi: a flexible and comprehensive Bioconductor package for the analysis of Infinium DNA methylation microarrays. Bioinformatics. 2014;30:1363-9.

[14] McCartney DL, Walker RM, Morris SW, McIntosh AM, Porteous DJ, Evans KL. Identification of polymorphic and off-target probe binding sites on the Illumina Infinium MethylationEPIC BeadChip. Genomics data. 2016;9:22-4.

[15] Zhou W, Laird PW, Shen H. Comprehensive characterization, annotation and innovative use of Infinium DNA methylation BeadChip probes. Nucleic acids research. 2017;45:e22.

[16] Team RC. R: A language and environment for statistical computing. Vienna, Austria: R Foundation for Statistical Computing; 2019.

[17] Lê S, Josse J, Husson F. FactoMineR: An R Package for Multivariate Analysis. Journal of Statistical Software. 2008;25:1-18.

[18] Phipson B, Maksimovic J, Oshlack A. missMethyl: an R package for analyzing data from Illumina's HumanMethylation450 platform. Bioinformatics. 2016;32:286-8.

[19] Hansen KD. IlluminaHumanMethylationEPICanno.ilm10b4.hg19: Annotation for Illumina's EPIC methylation arrays. 0.6.0 ed2017. p. R package.

[20] Kerr SM, Campbell A, Murphy L, Hayward C, Jackson C, Wain LV, et al. Pedigree and genotyping quality analyses of over 10,000 DNA samples from the Generation Scotland: Scottish Family Health Study. BMC medical genetics. 2013;14:38.

[21] Nagy R, Boutin TS, Marten J, Huffman JE, Kerr SM, Campbell A, et al. Exploration of haplotype research consortium imputation for genome-wide association studies in 20,032 Generation Scotland participants. Genome Med. 2017;9:23.

[22] McCarthy S, Das S, Kretzschmar W, Delaneau O, Wood AR, Teumer A, et al. A reference panel of 64,976 haplotypes for genotype imputation. Nat Genet. 2016;48:1279-83.

[23] Zeng Y, Amador C, Xia C, Marioni R, Sproul D, Walker RM, et al. Parent of origin genetic effects on methylation in humans are common and influence complex trait variation. Nature communications. 2019;10:1383.

[24] Haller T, Kals M, Esko T, Magi R, Fischer K. RegScan: a GWAS tool for quick estimation of allele effects on continuous traits and their combinations. Brief Bioinform. 2015;16:39-44.
